# A case report of pseudohypoaldosteronism type II with a homozygous *KLHL3* variant accompanied by hyperthyroidism

**DOI:** 10.1186/s12902-021-00767-w

**Published:** 2021-05-22

**Authors:** Rui Zhang, Simin Zhang, Yingying Luo, Meng Li, Xin Wen, Xiaoling Cai, Xueyao Han, Linong Ji

**Affiliations:** Department of Endocrinology and Metabolism, Peking University People’s Hospital, No 11, Xizhimen Nan Street, Xicheng District, 100044 Beijing, China

**Keywords:** Pseudohypoaldosteronism type II, Hyperkalemia, Secondary hyperparathyroidism, Hyperthyroidism, Case report

## Abstract

**Background:**

Pseudohypoaldosteronism type II (PHAII), also called Gordon syndrome, is a rare hereditary disease caused by variants in the *WNK1, WNK4, KLHL3* and *CUL3* genes. The combination of PHAII with hyperthyroidism and secondary hyperparathyroidism has not been reported previously.

**Case presentation:**

A 54-year-old female with recently diagnosed Graves’ disease presented hyperkalemia, hypertension, hypercalciuria, elevated levels of parathyroid hormone (PTH) and normal renal function. PHAII was established based on the finding of a homozygous variant (c.328 A > G, T110A) in the *KLHL3* gene. Low-dose thiazide diuretics normalized her potassium, calcium and PTH.

**Conclusions:**

PHAII caused by a *KLHL3* variant can affect adults later in life. This diagnosis should be considered in patients with hypertension, consistent hyperkalemia, and normal eGFR and can be corrected by thiazides. The patient also had hyperthyroidism and secondary hyperparathyroidism. The latter was also corrected by thiazide treatment. The hyperthyroidism was assumed to be unrelated to PHAII.

## Background

Pseudohypoaldosteronism type II (PHAII) is a rare hereditary disease characterized by hypertension, hyperkalemia and normal renal function. It is also called Gordon syndrome or familial hyperkalemic hypertension [1]. PHAII could be caused by variants in the *With no Lysine Kinases 1 (WNK1), WNK4, Kelch-like 3 (KLHL3)* and *Cullin 3 (CUL3)* genes [2]. These variants commonly lead to increased levels of WNK1/WNK4 protein in distal convoluted tubule (DCT) cells and affect the reabsorption of urinary sodium through the thiazide-sensitive NaCl cotransporter (NCC), resulting in volume expansion and the compensatory reduction of aldosterone secretion [3, 4]. Therefore, thiazide diuretics are effective in PHAII.

Graves’ disease is one of the most common causes of hyperthyroidism and is caused by an increase in anti-TSH-receptor antibody (TRAb) levels [5]. Hyperthyroidism can sometimes lead to hypokalemia and hypokalemic periodic paralysis by driving potassium into cells [6]. In addition, hyperthyroidism is commonly associated with hypercalciuria, but parathyroid hormone (PTH) levels are usually normal or low [7].

Here, we report a case of PHAII with a homozygous *KLHL3* gene mutation. The patient also had Graves’ disease and elevated PTH levels. To the best of our knowledge, this is the first case report of an adult with PHAII in combination with hyperthyroidism and hyperparathyroidism. We discussed the pathophysiological mechanism associating with the three disorders.

## Case presentation

A 54-year-old Chinese female came to our hospital because of hyperthyroidism and persistent hyperkalemia as well as an elevated PTH level in October 2018. She first presented with palpitations, fatigue, tremor, and weight loss in August 2017. She visited local doctors and was diagnosed with hyperthyroidism (Graves’ disease) with elevated total thyroxine (TT4), total triiodothyronine (TT3), free thyroxine (FT4), free triiodothyronine (FT3), decreased thyrotropin (TSH), and elevated anti-TSH-receptor antibody (TRAb) and an ultrasound presentation of abundant blood flow in the thyroid. Her serum potassium was 5.48 mmol/L at that time but was not noticed by the doctor. Thiamazole 15 mg daily was given. The patient felt better after the treatment, and the dose of thiamazole was reduced to 10 mg daily. Thyroid function steadily improved during the treatment (Table 1). However, her serum potassium was still elevated, between 5.3 and 5.8 mmol/L. She felt dizzy, with blood pressure measured as 167/94 mmHg in August 2018. She was diagnosed with hypertension, and a combination treatment of metoprolol 25 mg twice a day, nifedipine 30 mg twice a day, and furosemide 40 mg daily was prescribed to her to treat her hyperkalemia at the same time. However, her blood pressure was not well controlled, although she was in good compliance with medication. The serum potassium was still between 5.7 and 5.9 mmol/L.

**Table 1:** Thyroid function during treatment

	2018-3 (at the diagnosis of hyperthyroidism)	Normal range in local clinic	2018-11-09 (before thiazide)	2018-11-20 (after thiazide)	2019-2-11	2019-6-11	Normal range in our hospital
TT4 (µg/dl)	18.64	5.1–14.1	7.1	7.0			3.2–12.6
TT3 (ng/dl)	328.2	80–200	110.07	126.29			60–180
FT4 (pmol/L)	5.7	0.93–1.7	15.58	15.97	16.43	16.3	11.45–23.17
FT3 (pmol/L)	13.46	2.0-4.4	5.33	5.56	5.68	4.57	3.5–6.5
TSH (µIU/ml)	<0.005	0.27–4.2	0.008	0.005	0.004	0.097	0.55–4.78

Abbreviations: *TT4* total thyroxine, *TT3* total triiodothyronine, *FT4* free thyroxine, *FT3* free triiodothyronine, *TSH* thyroid-stimulating hormone

In one of the routine checks of hyperthyroidism in October 2018, her serum PTH was 123.6 pg/ml (normal reference range less than 88 pg/ml). Then, she visited our clinic, and presented with serum Ca^+^ at 2.39 mmol/L (normal range 2.2–2.65 mmol/L). PTH was 108.62 pg/ml (normal range less than 88 pg/ml). Twenty-four-hour urinary calcium was 9.52 mmol/day (normal reference range 5.9–6.5 mmol/day), serum K^+^ was 6.18 mmol/L, Na^+^ was 139 mmol/L, Cl^−^ was 108.6 mmol/L, and total CO_2_ was 21.2 mmol/L.

She was admitted to the ward of our department at that time. Physical examination showed that her blood pressure was 162/80 mmHg, her body mass index (BMI) was 29.8 kg/m^2^, and her thyroid was enlarged and soft. Further laboratory evaluation still showed elevated potassium and PTH with normal to low serum calcium (Table 2). The urinary calcium was normal in the second test, and arterial blood gas showed metabolic acidosis (Table 2). Biochemical bone markers showed increased bone alkaline phosphatase (ALP) and tartrate resistant acid phosphatase-5b (TRAP-5b), suggesting increased bone turnover. 25-Hydroxyvitamin D was 16.04 nmol/L (normal range 75–250 nmol/L). Her serum creatinine was 57 µmol/L, and the estimated glomerular filtration rate (eGFR) was 101.24 ml/min*1.73m^2^. Her serum cortisol and ACTH were normal, plasma renin was low or normal, and aldosterone was normal (Table 3). Abdominal ultrasound showed normal images of the pancreas and adrenal gland.

**Table 2:** Change of electrolytes before and after thiazide

	Before thiazide	After thiazide	Normal range
K^+^ (mmol/l)	5.20–5.44	4.81–5.22	3.50–5.30
Na^+^ (mmol/l)	138.2-138.8	135.0-138.8	137.0-147.0
Cl^−^ (mmol/l)	107.1-107.4	101.2–106.0	99.0-110.0
Mg^2+^ (mmol/l)	0.85		0.7–1.05
Ca^+^ (mmol/l)	2.09–2.38	2.36–2.46	2.20–2.65
P (mmol/l)	1.33–1.36	1.17–1.20	0.81–1.45
PTH (pg/ml)	119.54	69.18	12.0–88.0
pH value	7.326		7.35–7.45
HCO_3_^−^ (mmol/l)	21.0		22–27
BE (mmol/l)	-4.0		-3.0-3.0
AG (mmol/l)	14.4		8–12
Urinary K^+^ (mmol/day)	48.72		25–100
Urinary Na^+^ (mmol/day)	154.0		130–260
Urinary Ca^+^ (mmol/day)	5.82–6.90	5.90–6.50	2.50–7.50
Urinary P (mmol/day)	23.79–25.72	42.62–45.09	12.90–42.00

Abbreviations: *PTH* parathyroid hormone, *BE* base excess, *AG* anion gap

**Table 3:** Renin and aldosterone levels in this case

	Supine position	Normal range of supine position	Posture position 1	Posture position 2	Posture position 3	Normal range of posture position
Renin (µIU/ml)	2.7	2.8–39.9	4.2	3.7	9.5	4.4–46.1
Aldosterone (ng/dl)	4.5	3.0-23.6	20.5	6.0	7.8	3.0-35.3

The patient was previously healthy and did not have a family history of hyperkalemia. Her grandmother had been diagnosed with hypertension. Her parents were not consanguineous. Her father died of lung cancer, her mother and brother were healthy, and she did not have any children.

**Fig. 1:**
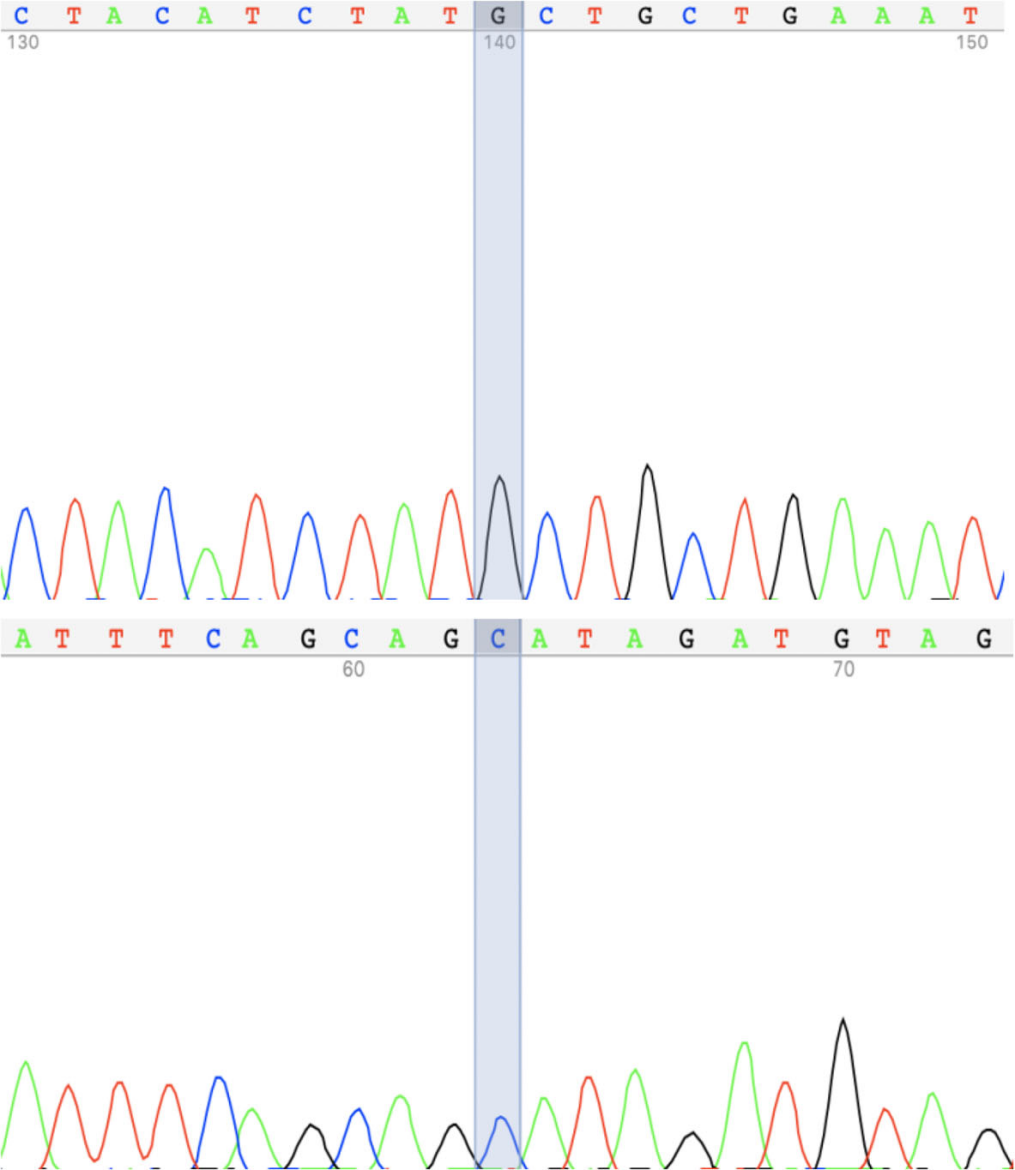
A homozygous missense variant c.328 A > G (T110A) in the KLHL3 gene by Sanger-Sequencing

Whole-exome sequencing (WES) of this patient was performed with a BGISEQ-500 platform (Beijing Genomics Institute, Shenzhen, China). A homozygous missense variant in the *KLHL3* gene, c.328 A > G (T110A) (NM_01745.3), was identified. The variant was then verified by Sanger sequencing (Fig. 1). The primers for Sanger sequencing were as follows. KLHL3-4 F: AGACAGGGCAGGAGACCATC, KLHL3-4R: AAAATGGTGGGTCCTGAGTG. This variant has not been reported before and does not exist in the ExAC database. The variant was predicted to be likely pathogenic according to the criteria of the American College of Medical Genetics (ACMG) and damaging according to other software programs (SIFT, PolyPhen-2, PROVEAN, CADD, and MutationTaster).

Furosemide 40 mg was stopped and changed to hydrochlorothiazide 25 mg daily, and metoprolol and nifedipine were continued. Two days later, her potassium decreased to a normal level (4.87 mmol/L). After discharge from the hospital, her serum potassium remained normal (4.81–5.08 mmol/L) at follow-up. Her blood pressure was well controlled, under 145/80 mmHg. PTH decreased to the normal range, and serum and urinary calcium were normal (Table 1).

## Discussion and conclusions

PHAII is a rare type of monogenic hypertension characterized by hyperkalemia, metabolic acidosis, hypercalciuria and otherwise normal renal function. It is also described as the opposite of Gitelman syndrome, which presents with hypokalemia, metabolic alkalosis, hypocalciuria and hypomagnesemia [8]. Increased reabsorption of urinary sodium through NCCs in DCT cells is the main pathophysiological mechanism of PHAII. Decreased urinary potassium excretion is secondary to increased Na^+^ reabsorption and leads to hyperkalemia and metabolic acidosis. As a result of hyperkalemia and volume expansion, the renin level is low, and aldosterone is normal or low [4].

Variable mutations in genes including *WNK1, WNK4, KLHL3* and *CUL3* are reported to be causative for PHAII [2]. WNK (especially WNK4) regulates the activities of NCC, the epithelial Na^+^ channel ENaC, and the K^+^ channel ROMK in the DCT. Among them, NCC has a crucial role in the development of PHAII. In addition, WNK is controlled through ubiquitination by KLHL3/CUL3. The KLHL3 protein recruits substrates for CUL3-based ubiquitin ligase complexes and downregulates NCC expression [3]. KLHL3 knockout mice exhibit PHAII-like phenotypes [9]. Hypercalciuria in PHAII may occur due to decreased calcium reabsorption in DCT. The expression of epithelial Ca^+^ channels in the DCT, including transient receptor potential channel vanilloid subtype 5 (TRPV5), TRPV6, and calbindin-D28k (CBP-D28k), is involved in the mechanism. Animal studies found that mutations in *WNK4* downregulate TRPV6 and CBP-D28k and lead to decreased calcium reabsorption [10].

In this case, the patient presented with typical features, including hyperkalemia, hypertension, hypercalciuria and low renin levels, and all of these irregularities were restored to normal by low-dose thiazide diuretics. However, some distinctive features that have not been described in the literature were present in this case. First, a persistently elevated PTH level was discovered and corrected by thiazide diuretics. Second, the patient had hyperthyroidism (Graves’ disease). These two features made the diagnostic process more challenging.

Hyperthyroidism can sometimes cause hypokalemia and hypokalemic periodic paralysis by driving potassium into cells, which often occurs among young male patients[6]. Hyperthyroidism can also contribute to hypercalciuria and occasionally hypercalcemia, but PTH levels remain normal or low[7]. Nevertheless, hyperkalemia and increased PTH cannot be explained by hyperthyroidism. Other possible etiologies were considered in the differential diagnosis. Two major causes of hyperkalemia are increased potassium release from cells and reduced urinary potassium excretion. The latter is the most common cause of persistent hyperkalemia. The patient had a normal eGFR and did not use any potassium-sparing diuretics, angiotensin converting enzyme inhibitors or nonsteroidal anti-inflammatory drugs, which can reduce urinary potassium excretion. After the confirmation of PHAII with genetic analysis, we searched previous literature but did not find a combination of hyperthyroidism and PHAII. Thus, we speculate that these two disorders may be unrelated.

Another feature of this case was a persistently elevated PTH level. Although increased urinary calcium can be explained by hyperthyroidism or the use of furosemide [7], the elevated PTH was inconsistent with this diagnosis. The low level of 25-hydroxyvitamin D obscured the cause of secondary hyperparathyroidism in this case. Finally, the normalization of calcium and PTH with only thiazides and without vitamin D supplementation excluded the possibility of secondary hyperparathyroidism due to vitamin D deficiency. Thus, we believe that the increase in PTH may be secondary to hypocalcemia as a result of the increased excretion of urinary calcium in PHAII, although a change in PTH in PHAII was not described previously.

PHAII is a hereditary disease, but it may develop at an advanced age. In a cohort study of 52 kindreds including 126 PHAII patients, both dominant and recessive *KLHL3* mutations could cause PHAII [2]. The phenotypic severity varies among PHAII patients with different gene mutations. Subjects with *CUL3* mutations present at a much younger age and have more severe hyperkalemia and metabolic acidosis. In contrast, patients with *KLHL3* mutations may develop hypertension and other symptoms at a later age. In this patient, we found a homozygous c.328 A > G (T110A) variant in *KLHL3* that has not been found in previous literature.

**Fig. 2:**
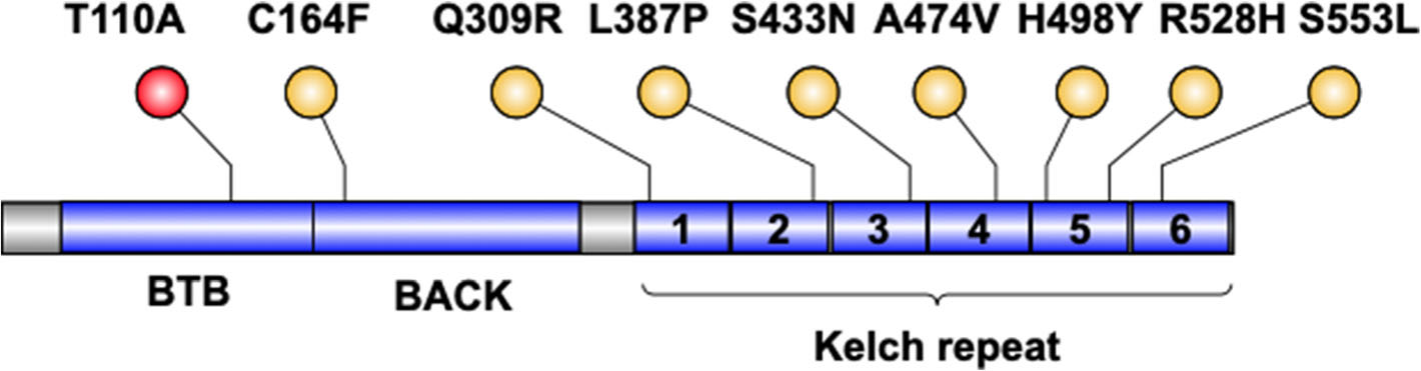
The structure of KLHL3 protein and the reported mutation sites

Different sites of *KLHL3* gene mutations, including H498Y, L387P, C164F, S433N, Q309R, R528H, A474V, and S553L, were previously reported in patients with PHAII [11–17]. The KLHL3 protein contains three domains: a BACK (BTB and C-terminal Kelch) domain, an N-terminal BTB (Broad-Complex, Tramtrack and Bric a brac) domain, and a six-bladed β-propeller structure formed from Kelch-like repeats (Fig. 2) [18]. The T110A mutation of the present case is in the BTB domain, which has a role in binding to the CUL3 protein. The mutations, including the present case and those in the previous literature, are summarized in Table 4; Fig. 2. Both heterozygous and homozygous mutations were described. The ages of the affected patients ranged from 1 month to 69 years. All of them had typical clinical features, including hypertension and hyperkalemia.

**Table 4:** Review of the characteristics of PHAII patients with KLHL3 gene mutations

Author	Mutation	Exon	het/hom	Pattern of inheritance	Family history or not	Age at diagnosis (years)	Sex	Serum potassium
This study	T110A	4	hom	recessive	sporadic	56	M	5.44
Yang [16]	S433N	11	het	dominant	sporadic	23	F	6.3
Doan [17]	A474V	11	het	dominant	sporadic	0.16	F	6.4
Kliuk-Ben Bassat [13]	S553L	13	hom	recessive	pedigree	1	M	7.1
						34	F	6.2
	Q309R	8	het	dominant	pedigree	63	M	5.6
						62	F	4.8
						57	M	5.1
						54	M	6.4
						47	F	5.1
						47	F	5.1
						44	M	NA
						18	M	5.2
						15	F	5.8
						12	F	5.3
Mayan [12]	Q309R	8	het	dominant	pedigree	69	F	5.4
						48	F	5.4
						11	M	6.4
						11	M	7.2
						10	F	5.3
	R528H	13	het	dominant	pedigree	38	F	5.6
						15	F	6
						13	F	6.2
						8	F	5.8
						2	M	7
Park [11]	C164F	4	het	dominant	sporadic	0.8	F	6.3
	S433N	11	het	dominant	pedigree	1.7	F	6.9
	S433N	11	het	dominant	pedigree	23	F	6.4
Mitani [14]	L387P	9	het	dominant	sporadic	3	M	6.6
Kelly [15]	H498Y	13	het	dominant	pedigree	18	M	7.3

This patient was diagnosed at the age of 54. The absence of the phenotype in her parents and siblings indicates that this variant may be recessive. However, we cannot confirm this hypothesis without pedigree research. Most previous case reports of PHAII were in children or young adults, but there have been adults who were diagnosed later in life[12, 13]. It is not clear why some mutations of *KLHL3* can cause PHAII at an advanced age. It is possible that the phenotype of these mutations is milder; functional studies are needed to confirm this hypothesis. Nevertheless, this case highlights the need to consider hereditary diseases in adults and establish the diagnosis by exome sequencing.

In conclusion, we report a case of a middle-aged Chinese woman with PHAII caused by a novel homozygous variant in *KLHL3*. This is the first case report of PHAII in combination with hyperthyroidism and secondary hyperparathyroidism. We assumed that the hyperparathyroidism was secondary to PHAII and was not associated with the mutation. This rare case reflected the complexity in the differential diagnosis of electrolyte disorders and indicated the importance of genetic analysis in the diagnosis of a hereditary disease.

## Data Availability

Data sharing is not applicable to this article as no datasets were generated or analyzed during the current study.

## Abbreviations

PHAII: Pseudohypoaldosteronism type II
WNK1: With no lysine kinases 1
WNK4: With no lysine kinases 4
KLHL3: Kelch-like 3
CUL3: Cullin 3
DCT: Distal convoluted tubule
NCC: Thiazide-sensitive NaCl cotransporter
PTH: Parathyroid hormone
TSH: Thyrotropin
TRAb: Anti-TSH-receptor antibody
BMI: Body mass index
ALP: Bone alkaline phosphatase
TRAP-5b: Tartrate resistant acid phosphatase-5b
TRPV5: Transient receptor potential channel vanilloid subtype 5
TRPV6: Transient receptor potential channel vanilloid subtype 6
CBP-D28k: Albindin-D28k
WES: Whole exome sequencing
